# Investigation of 5’-Norcarbocyclic Nucleoside Analogues as Antiprotozoal and Antibacterial Agents

**DOI:** 10.3390/molecules24193433

**Published:** 2019-09-21

**Authors:** Anastasia L. Khandazhinskaya, Elena S. Matyugina, Pavel N. Solyev, Maggie Wilkinson, Karen W. Buckheit, Robert W. Buckheit, Larisa N. Chernousova, Tatiana G. Smirnova, Sofya N. Andreevskaya, Khalid J. Alzahrani, Manal J. Natto, Sergey N. Kochetkov, Harry P. de Koning, Katherine L. Seley-Radtke

**Affiliations:** 1 Engelhardt Institute of Molecular Biology of the Russian Academy of Sciences, 32 Vavilov St., Moscow 119991, Russia; matyugina@gmail.com (E.S.M.); solyev@gmail.com (P.N.S.); kochet@eimb.ru (S.N.K.); 2 ImQuest BioSciences, 7340 Executive Way Suite R, Frederick, MD 21704, USA; mwilkinson@imquestbio.com (M.W.); kbuckheit@imquestbio.com (K.W.B.);; 3 Central Tuberculosis Research Institute, 2 Yauzskaya Alley, Moscow 107564, Russia; lchernousova@mail.ru (L.N.C.); s_tatka@mail.ru (T.G.S.); andsofia@mail.ru (S.N.A.); 4 Institute of Infection, Immunity and Inflammation, University of Glasgow, Sir Graeme Davies Building, 120 University Place, Glasgow G12 8TA, UK; Khalid-zh@hotmail.com (K.J.A.); Manal.Natto@glasgow.ac.uk (M.J.N.); harry.de-koning@glasgow.ac.uk (H.P.d.K.); 5 Department of Clinical Laboratory, College of Applied Medical Sciences, Taif University, Taif 21974, Saudi Arabia; 6 Department of Chemistry & Biochemistry, University of Maryland, Baltimore County, 1000 Hilltop Circle, Baltimore, MD 21250, USA

**Keywords:** carbocyclic, nucleosides, tuberculosis, leishmanial, antiparasitic, mycobacterial

## Abstract

Carbocyclic nucleosides have long played a role in antiviral, antiparasitic, and antibacterial therapies. Recent results from our laboratories from two structurally related scaffolds have shown promising activity against both *Mycobacterium tuberculosis* and several parasitic strains. As a result, a small structure activity relationship study was designed to further probe their activity and potential. Their synthesis and the results of the subsequent biological activity are reported herein.

## 1. Introduction

**Figure 1:**
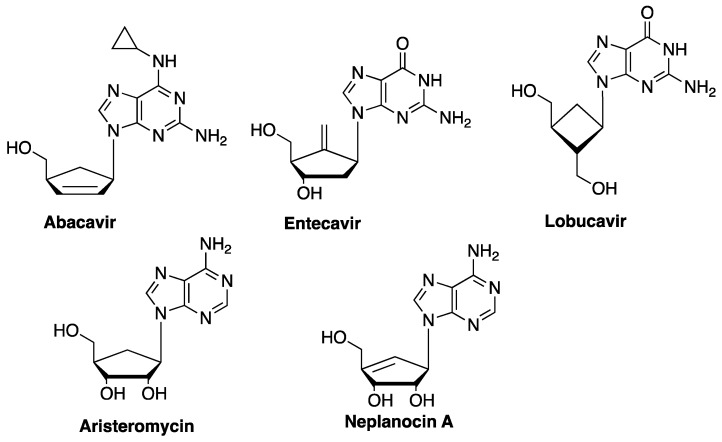
Some historically important carbocyclic nucleosides.

Carbocyclic nucleoside analogues, compounds in which a methylene group replaces the oxygen atom in the furanose sugar moiety, have a distinguished history as anti-infectious agents, including the Food and Drug Administration (FDA)-approved antiviral drugs abacavir, entecavir, and lobucavir, as well as the naturally occurring neplanocin and aristeromycin (Figure 1) [1,2]. 5′-Norcarbocyclic nucleoside analogues were originally created as inhibitors of *S*-adenosylhomocysteine hydrolase (SAHase), a cellular enzyme involved in many biological methylations [3].

**Figure 2:**
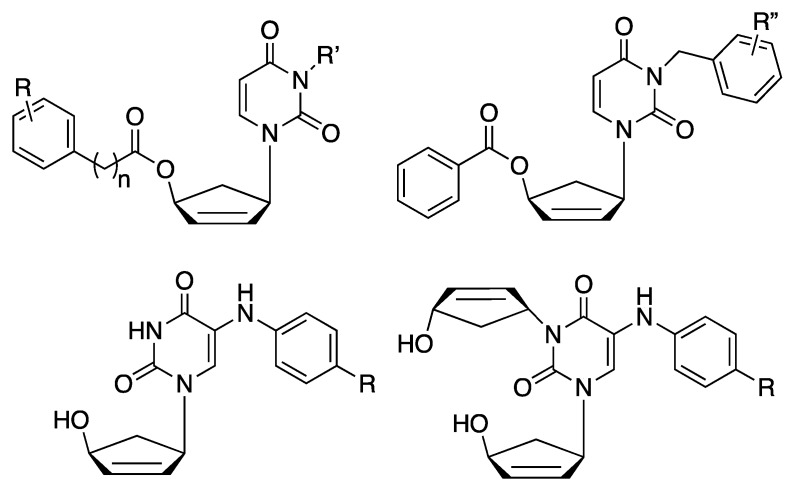
Previous leads.

Recently it was reported that 5′-norcarbocyclic nucleoside analogues can serve as Human Immunodeficiency Virus (HIV) non-nucleoside reverse transcriptase inhibitors (NNRTIs) with Ki’s of 5–19 µM for wild type and 1–55 µM for some mutant strains of HIV reverse transcriptase (Figure 2) [4,5]. Other compounds from this group also proved to be potent inhibitors of *Mycobacterium tuberculosis* H37Rv with Minimum Inhibitory Concentration (MIC) values between 10 and 40 µg/mL and the multi-drug resistant (MDR) strain MS-115 with MIC values ranging from 5 to 20 µg/mL (Figure 2) [6,7]. In both cases the 5′-norcarbocyclic nucleosides exhibited activity against resistant strains.

**Figure 3:**
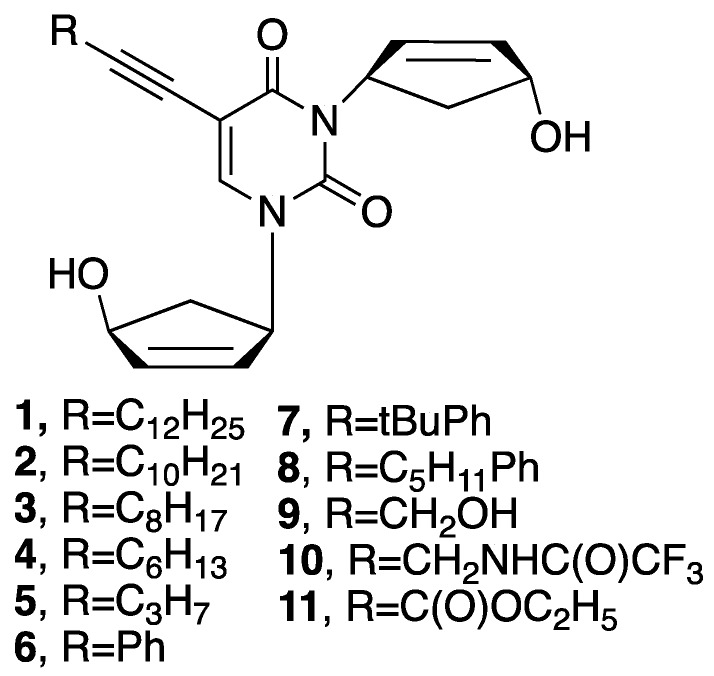
Target compounds.

More recently 5′-norcarbocyclic nucleoside analogues were screened against the major kinetoplastid pathogens *Trypanosoma* and *Leishmania* (Figure 3) [8]. Among the tested compounds the most active proved to be 5-tetradecinyluracil possessing two 5'-norcarbocyclic moieties at the N1 and N3 positions of the pyrimidine base (1). Compound 1 exhibited equivalent activity against both pathogens with an EC_50_ of 8.0–11.9 µM, and was effective against resistant forms of these pathogens (both resistant to other pyrimidines and to first-line clinical drugs). This rendered it an interesting lead for further structural optimization. The strong correlation between the trypanocidal and anti-leishmanial activities indicated that this mechanism is likely shared between the two species. 

## 2. Results

### 2.1. Chemistry

As a result of these observations, a series of 5'-norcarbocyclic uracil derivatives with various 5-alkynyl or 5-arylalkynyl substituents (Figure 3) were designed relative to the parent compound **1**, in an effort to explore their potential biological activity and function.

**Scheme 1:**
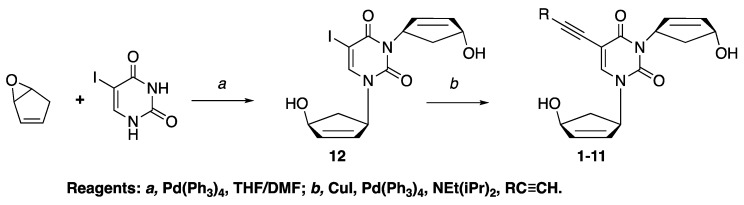
Synthesis of the target compounds.

Conveniently, compound **12**, the key intermediate for the target products synthesis (Scheme 1), was previously reported as side product [9]**.** As a result, we used the same method of synthesis, but enhanced an excess of epoxy-cyclopentene (3 mol eqv instead of 1.3) to improve the yield. Unlike many synthetic routes to realize carbocyclic nucleosides [10,11], the synthesis of the compounds was straightforward and followed a route previously reported from our group [7,9]. Products **1**–**11** were obtained as racemic mixtures, in good yields, but the presence of a large number of minor impurities in the reaction mixture forced us to consistently use two different chromatographic systems. All target compounds were verified for structural accuracy and purity using spectroscopic and analytical methods as described in the experimental section. 

### 2.2. Biological Evaluation

The results of the initial screening against *T. brucei* and *Leishmania* strains are shown in Table 1 and Table 2, respectively. Against *T. brucei*, the highest activity was observed for compound **2** (R = decyl), with an EC_50_ of 3.1 +/−0.1 µM, followed by **7** and **8** (R = 4-tBu-phenyl and 4-pentylphenyl, EC_50_ of ~8 µM each), **3** and **4** (R = octyl and hexyl, EC_50_ of ~13 µM each) (Table 1). It thus appears that long aliphatic R chains (C_10_, C_12_) have the best antitrypanosomal activity, with C_8_ and C_6_ still displaying EC_50_ values < 15 µM, whereas short or aromatic R chains (C_2_, C_3_, Ph) or polar substituents (in compounds **10**, **11**) displayed little or no activity at the highest concentration tested. In order to probe whether their mode of action depended on uracil metabolism, we tested the compounds in parallel against a pyrimidine auxotrophic cell line that is hypersensitized to pyrimidine antimetabolites such as 5-fluorouracil (5FU) (PYR6-5 ^-/-^) [12] and against a 5FU-resistant strain (5FURes) [13]. Several of the test compounds displayed a minor but significant reduction in trypanocidal activity against both of these strains (all <2-fold), whereas sensitization or resistance to 5FU was as much as 37.4- and 28.7-fold for the PYR6-5^-/-^ and 5FURes strains, respectively. Similarly, some of the compounds displayed slightly higher EC_50_ values against the multi-drug resistant strain B48 (<1.5-fold), which is insignificant compared to the 219-fold resistance to pentamidine.

The cytotoxicity of compounds **1**–**11** was estimated using the U-937 laboratory adapted monocytic cell line (Table 1). Nonoxynol-9 was also evaluated in parallel as a positive control and was toxic at the expected concentration. While compounds **1**, **2**, and **4** exhibited some promising selectivity, the toxicity of the remaining compounds was similar to or greater than their activity.

Most of the compounds displayed a moderate effect against *L. mexicana* promastigotes, in the range 10–30 µM. The same compounds that lacked activity against *T. brucei* were ineffective against *L. mexicana*, and also displayed the least activity against *Trichomonas vaginalis*. Compound **9** (R = hydroxymethyl) was much less active than **2**–**4**, **7**, and **8** against *T. brucei* and *Trichomonas vaginalis*, and did not show any antileishmanial activity at all. Compounds **5** (R = propyl), **6** (R = phenyl), **10** (R = trifluoroacetamidomethyl), and **11** (R = ethyloxycarbonylmethyl) were completely inactive against all strains.

The fact that short or polar R chains displayed poor activity may reflect a dependence on hydrophobic properties to cross the parasite plasma membrane. We note that all inactive compounds (**5**, **6**, **9**, **10**, **11**) have cLogP values below 2 whereas the active ones had cLogP values in the range 2.61–4.37 (ChemDraw Pro 10.0, CambridgeSoft, PerkinElmer, Waltham, MA, USA). 

As with *T. brucei*, there were significant but only minor (<2.2-fold) differences in EC_50_ values between wild-type and a 5FU-resistant strain (Lmex5FURes), compared with a >400-fold resistance to 5FU. We propose that this compound class displays general anti-kinetoplastid activity that likely depends on passive diffusion and does not principally act on pyrimidine transport or metabolism. The activity is relatively specific in that none of the compounds displayed EC_50_ values for *T. vaginalis* under 30 µM.

In order to determine the lead compound(s) prior to evaluation against the virulent strains of *M. tuberculosis*, all the 11 compounds were initially evaluated for inhibition of an attenuated strain of *M. tuberculosis* ATCC 25,177 (H37Ra), which is closely related to the virulent laboratory strain H37Rv (Table 3).

Compounds **1**–**4**, **7**, and **8**, displaying better effects against the attenuated strain, were selected for further screening against two virulent strains of *M. tuberculosis*, a laboratory strain *M. tuberculosis* H37Rv and a clinical strain *M. tuberculosis* MS-115 (Table 4) with multiple drug resistance. The antimycobacterial effect was studied by measuring the growth dynamics of strains of *M. tuberculosis* in the enriched medium Middlebrook 7H9 in the automated growth registration system BACTEC MGIT 960. Different concentrations of the compounds were compared to the growth of strains on media not containing preparations and media containing control preparations at critical concentrations (rifampicin 1 μg/mL, isoniazid 0.1 μg/mL, and levofloxacin 1.5 μg/mL). Each of the concentrations of the tested compounds as well as control samples were assayed in triplicate. In all positive samples, the culture of mycobacteria of the tuberculosis complex was confirmed to grow and there was no contamination with nonspecific microflora. When exposed to control preparations at critical concentrations, growth in the sensitive culture was not observed, and the growth parameters of the MDR strain, when incubated with isoniazid and rifampicin, did not differ from the control without the drug.

The antibacterial activity of the synthesized compounds was also evaluated against different other bacterial species, both Gram-positive (*Staphylococcus aureus* NRS384, *Mycobacterium smegmatis* (*M. smegmatis*) 700,084, *Mycobacterium bovis* (*M. bovis*) 35,737) and Gram-negative (*Pseudomonas aeruginosa* ATCC 27,853 and *Escherichia coli* ATCC 25,922). Most of the bacteria were not sensitive to the tested compounds at concentrations up to 250 µM. The exception was *M. smegmatis* 700,084, the growth of which was completely inhibited by compounds **7** and **8** at concentrations of 250 µM. Outside this single finding, none of the compounds exhibited a MIC against any of the strains, however a decrease in growth of *S. aureus* NRS384 in the presence of compounds **10**–**11** was noted; this gave rise to an extrapolated IC_50_ of approximately 100 µM.

## 3. Discussion

Infectious diseases could be said to be making a come-back, due to chronic under-investment in anti-infective drug development, reduced acceptance of vaccines, and the increasing spread and severity of drug resistance [14,15]. Most antibacterial, antifungal, and antiparasitic drugs are decades old, and the continued lack of development threatens the treatability of many infectious diseases. Even when new therapies are proposed they are often developed from existing antimicrobial agents, e.g., new penicillins, new tetracyclines, diamidines, new minor groove binders etc. [16,17,18,19]. While such strategies can (temporarily) circumvent resistance, it was inherent to this approach that resistance to the class of compound was already widespread in the microbial populations targeted. 

Here we report on the antimicrobial activities of a very novel class of compounds and test for reduced activity against pathogen strains with acquired resistance to important antibiotic and antiparasitic drugs. For *T. b. brucei* we utilized a panel of indicative strains for which EC_50_ values were determined in parallel with the wild-type sensitive strain. The B48 line is highly resistant to many of the most potent trypanocides, including the diamidines (pentamidine, diminazene, furamidines) [20,21,22,23], melaminophenyl arsenicals (Melarsoprol, Cymelarsan) [20], and many nucleoside analogues [24,25], because of defects in the TbAT1/P2 and HAPT1/TbAQP2 drug transporters [26,27,28]. Indeed, drug resistance in protozoa is very often linked to transporter or other uptake defects [29,30,31]. The current series of test compounds did not display notably reduced effectiveness against this multi-drug resistant line. In fact, the observations that the most effective test compounds are also the most hydrophobic, and that drug uptake likely proceeds through passive diffusion, would seem an advantage in avoiding many cross-resistance mechanisms. We can also rule out that the test compounds, despite having uracil at their core, are taken up by uracil transporters as both the *T. b. brucei* and *L. mexicana* 5FURes strains display much-reduced uptake of uracil and 5FU [12] and the kinetoplastid uracil transporters are known not to accept substrates with large substitutions on their 5-position, even methyl [32,33]. As the uracil transporters are not essential to these parasites and are easily lost or diminished under drug pressure [12,13], this serves to further highlight the importance of uptake through passive diffusion for this compound series. 

The pyrimidine auxotrophic cell line PYR6-5 is hypersensitive to pyrimidine antimetabolites because it is incapable of synthesizing its own pyrimidines, which would compete with the antimetabolite for the target enzyme(s) [13]. The lack of a clear difference in trypanocidal activity of the current test compounds against this strain and the wild-type control indicates that their mode of action is not any pyrimidine-dependent pathway but remains to be elucidated. None of the compounds displayed more than marginal activity against *T. vaginalis*, an unrelated protozoan parasite, or against a range of bacteria, showing that despite the probable cell entry by diffusion the cytotoxic affects were moderately species specific. 

The current study builds on our recent work on 5’-norcarbocyclic uridine analogues, extending, in particular, the structure-activity relationship (SAR) of the analogues by adding a substantial chain to the 5-position of the pyrimidine ring. In addition, we extended the study to include antibacterial activities. In the previous study, compound **1** was the most active, and in the current study a modest improvement was observed with **2** being more than twice as potent against *T. brucei*. More importantly, it became clear that there was a minimum chain length when the chain is aliphatic, or more likely, a minimum LogP [34], which likely was enabling passive diffusion into the cell. 

It appears that the actual type of side chain may not be important, but rather it may be its role in enabling the entry of the pharmacophore into the cell. Similarly, we have recently shown that by linking inhibitors of Trypanosome Alternative Oxidase (TAO) to lipophilic cations via a sufficiently long linker, this enabled better interactions with the target enzyme’s binding site [35,36]. This correlates with the results seen with our earlier study [8] thus extending the SAR of the pharmacophore by coupling to a suitable, hydrophobic side chain at the 5-position is appropriate. Moreover, using the triple bond may serve to keep the flexible side chain suitably clear of the pharmacophore. Moreover, since the compounds do not appear to act on pyrimidine-binding enzymes, future SAR efforts will include alterations to the functional groups of the uracil moiety, as well as to identify the drug target through induction of resistance followed by whole-genome sequencing, metabolomics, and cellular assays. This will in turn inform further SAR studies.

We anticipated strong activity for this series as antimycobacterial agents, as we have previously shown for the other 5’-norcarbocyclic nucleosides [6,7]. Unfortunately, this particular group of compounds showed antimycobacterial activity only at a concentration that exerts a cytotoxic effect on eukaryotic cells. Efforts are underway to expand the work on the synthesis of new compounds, which may exhibit a higher inhibitory effect against mycobacteria and lower cytotoxicity. The results of those studies will be reported in due course as they become available.

## 4. Materials and Methods

### 4.1. Chemistry Experimental

#### 4.1.1. General

The reactions were performed with the use of commercial reagents (Acros (Geel, Belgium), Aldrich (St. Louis, MO, USA), and Fluka (Bucharest, Romania)),); anhydrous solvents were purified according to the standard procedures. Column chromatography was performed on Silica Gel 60 0.040–0.063 mm (Merck, Darmstadt, Germany) columns. Thin layer chromatography (TLC) was performed on Silica Gel 60 F_254_ aluminum-backed plates (Merck, Darmstadt, Germany). Preparative layer chromatography (PLC) was performed on Silica Gel 60 F_254_ glass-backed plates (Merck, Darmstadt,Germany). NMR spectra were registered on an AMX III-400 spectrometer (Bruker, Newark, Germany) with the working frequency of 400 MHz for ^1^H NMR (Me_4_Si as an internal standard for organic solvents) and 100.6 MHz for ^13^C NMR (with carbon-proton interaction decoupling). 

High resolution mass spectra were measured on Bruker micrOTOF II instruments using electrospray ionization (ESI HRMS). The measurements were done in positive ion mode (interface capillary voltage 4500 V) in a mass range from *m/z* 50 to *m/z* 3000 Da; external or internal calibration was done with ESI Tuning Mix™ (Agilent Technologies, Santa Clara, CA, USA). Nitrogen was applied as a dry gas (6 L/min); the interface temperature was set at 180 °C, nebulizer pressure: 0.4 Bar; flow rate: 3 µL/min. Samples were injected into the mass spectrometer chamber from the Agilent 1260 HPLC system equipped with Agilent Poroshell 120 EC-C18 column (3.0 × 50 mm; 2,7 µm) and an identically packed security guard, using an autosampler. The samples were injected from acetonitrile (LC-MS grade) solution. The column temperature was 30 °C and 5 μL of the sample solution was injected. The column was eluted in a gradient of concentrations of A (acetonitrile) in B (water) with the flow rate of 400 µL/min in the following gradient parameters: 0–15% A for 6.0 min, 15–85% A for 1.5 min, 85–0% A for 0.1 min, 0% A for 2.4 min.

#### 4.1.2. Compound Synthesis and Characterization

*1-(4′-Hydroxy-2′-cyclopenten-1′-yl)-3-(4′′′-hydroxy-2′′′-cyclopenten-1′′′-yl)-5-iodouracil* (**12**). Epoxy-cyclopentene (1.2 g, 15 mmol) in freshly distilled tetrahydrofuran (THF) (15 mL) and tetrakis(triphenylphosphine)palladium (0) [Pd(PPh_3_)_4_] (0.87 g; 0.75 mmol) were added to a stirred solution of 5-iodouracil (1.2 g; 5 mmol) in anhydrous *N*,*N*-dimethylformamide (DMF) (100 mL). The reaction mixture was stirred at room temperature for 18 h. Purification on silica gel column eluting with CHCl_3_:MeOH (98:2) gave the product as off-white powder (724 mg, 1.8 mmol, 36%). ^1^H NMR (CDCl_3_): 1.62–1.54 (1H, m, Hb-5′), 1.94–1.88 (1H, m, Hb-5′′), 2.77–2.69 (1H, m, Ha-5′), 2.88–2.80 (1H, m, Ha-5′′), 3.07 (2H, br.s, HO-4′ and HO-4′′), 4.70–4.67 (1H, m, H-4′), 4.85–4.82 (1H, m, H-4′′), 5.53–5.49 (1H, m, H-1′), 5.72–5.69 (1H, m, H-3′), 5.81–5.78 (1H, m, H-3′′), 5.92–5.88 (1H, m, H-1′′), 6.11–6.08 (1H, m, H-2′), 6.26–6.24 (1H, m, H-2′′), 7.82 (1H, s, H-6). ^13^C NMR (CDCl_3_): 37.4, 40.5 (C-5′, C-5′′), 57.8, 60.6 (C-1′, C-1′′), 68.7 (C-5), 74.4, 76.2 (C-4′, C-4′′), 128.6, 130.6 (C-2′, C-2′′), 131.3, 132.0 (C-3′, C-3′′), 137.2 (C-6), 140.4, 144.8 (C-4, C-2).

*1-[4′-Hydroxy-2′-cyclopenten-1′-yl]-3-(4′′-hydroxy-2′′-cyclopent-en-1′′-yl)-5-tetradecynyluracil* (**1**). Compound **12** (80 mg; 0.2 mmol) was dissolved in freshly distilled DMF (15 mL), followed by addition of copper (I) iodide (13 mg; 0.1 mmol), *N,N*-diisopropyl ethylamine (139 µL; 0.8 mmol), Pd(PPh_3_)_4_ (23 mg; 0.02 mmol) and 1-tetradecyn (64 µL; 0.26 mmol). The reaction mixture was stirred at room temperature under argon atmosphere. The progress of the reaction was monitored by TLC in 95:5 CHCl_3_/MeOH. After 2 h, 0.3 mL of 0.5 M EDTA/H_2_O (pH 8.0) was added to the reaction mixture and then the mixture was concentrated in vacuo. The product was purified by column chromatography on a silica gel column eluting with 98:2 CHCl_3_/MeOH, followed by a second column eluting with EtOAc/hexane (4:1) to give the target compound as an off-white powder (66 mg, 70%). Rf 0.38 (eluting with CHCl_3_:MeOH (98:2)). ^1^H NMR (CDCl_3_) δ 1.22–1.19 (3H, m, CH_3_), 1.25 (16H, s, (CH_2_)_8_), 1.37–1.33 (2H, m, CH_2_-c), 1.50–1.49 (2H, m, CH_2_-b), 1.61–1.60 (1H, m, Hb-5′), 1.92–1.90 (1H, m, Hb-5′′), 2.30–2.28 (2H, m, CH_2_-a), 2.72–2.68 (1H, m, Ha-5′), 2.93–2.89 (1H, m, Ha-5′′), 3.98–4.01 (1H, m, OH) 4.67–4.62 (1H, m, OH), 4.82–4.80 (1H, m, H-4′), 5.50–5.48 (1H, m, H-4′′′), 5.67–5.65 (1H, m, H-1′), 5.77–5.75 (1H, m, H-3′), 5.84–5.83 (1H, m, H-3′′), 5.87–5.85 (1H, m, H-1′′), 6.06–6.04 (1H, m, H-2′), 6.20–6.18 (1H, m, H-2′′), 7.47 (1H, s, H-6). ^13^C NMR (CDCl_3_) δ 14.09 (CH_3_), 19.62, 22.67, 28.61, 29.15, 29.34, 29.63 (11C, C-5′), 31.91 (C-5′′), 37.31 (C-4′), 40.49 (C-1′), 56.77 (C-1′′), 60.49 (C-4′′), 71.19, 74.68 (C≡C), 95.20 (C-5), 130.73 (C-2′, C-2′′), 131.48 (C-3′, C-3′′), 137.13 (C-6), 141.42, 139.87 (C-4, C-2). HRMS: found *m/z* 469.3059; calcd for C_28_H_40_N_2_O_4_ [M + H]^+^ 69.3061. 

*1-[4′-Hydroxy-2′-cyclopenten-1′-yl]-3-(4′′-hydroxy-2′′-cyclopent-en-1′′-yl)-5-dodecynyluracil***(2).** Compound **2** was obtained similarly to the procedure described for **1**, with 1-dodecyne as a terminal alkyne. Purification (CHCl_3_/MeOH 98:2 then 95:5) and repurification (EtOAc/hexane 1:2) by PLC on silica gel afforded the title compound **2** as a white powder (51 mg, 58%) ^1^H NMR (CDCl_3_): 0.91–0.82 (3H, m, CH_3_), 1.29–1.27 (12H, m, (CH_2_)_6_), 1.39–1.36 (2H, m, CH_2_-c), 1.60–1.55 (2H, m, CH_2_-b), 1.70–1.60 (1H, m, Hb-5′), 2.02–1.94 (1H, m, Hb-5′′), 2.40–2.35 (2H, m, CH_2_-a), 2.76–2.68 (1H, m, Ha-5′), 2.92–2.79 (1H, m, Ha-5′′), 4.74–4.72 (1H, m, H-4′), 4.90–4.88 (1H, m, H-4′′), 5.58–5.54 (1H, m, H-1′), 5.76–5.73 (1H, m, H-3′), 5.85–5.83 (1H, m, H-3′′), 5.94–5.91 (1H, m, H-1′′), 6.14–6.12 (1H, m, H-2′), 6.29–6.26 (1H, m, H-2′′), 7.56 (1H, s, H-6). ^13^C NMR (CDCl_3_): 14.1 (CH_3_), 19.6, 22.6, 28.6, 29.0, 29.1 × 2, 29.5, 29.6, 31.9 ((CH_2_)_9_,), 37.3, 40.3 (C-5′, C-5′′), 56.8, 60.5 (C-1′, C-1′′), 71.4 (alkyne^α^), 74.6, 76.3 (C-4′, C-4′′), 95.2 (^β^alkyne), 100.8 (C-5), 130.7, 131.3 (C-2′, C-2′′), 137.1, 140.0 (C-3′, C-3′′), 141.65 (C-6), 150.6, 162.1 (C-4, C-2). HRMS: found *m/z* 441.2755; calcd for C_26_H_36_N_2_O_4_ [M + H]^+^ 41.2748.

*1-[4′-Hydroxy-2′-cyclopenten-1′-yl]-3-(4′′-hydroxy-2′′-cyclopent-en-1′′-yl)-5-decynyluracil* (**3**). Compound **3** was obtained similarly to the procedure described for **1**, with 1-decyne as a terminal alkyne. Purification (CHCl_3_/MeOH 98:2 then 95:5) and repurification (EtOAc/hexane 1:2) by PLC on silica gel afforded the title compound **3** as a white powder (65 mg, 79%). ^1^H NMR (CDCl_3_): 0.86–0.82 (3H, m, CH_3_), 1.25–1.23 (8H, m, (CH_2_)_4_), 1.36–1.29 (2H, m, CH_2_-c), 1.55–1.47 (2H, m, CH_2_-b), 1.70–1.60 (1H, m, Hb-5′), 1.97–1.90 (1H, m, Hb-5′′), 2.34–2.28 (2H, m, CH_2_-a), 2.76–2.71 (1H, m, Ha-5′), 2.89–2.79 (1H, m, Ha-5′′), 4.69–4.67 (1H, m, H-4′), 4.84–4.82 (1H, m, H-4′′), 5.53–5.51 (1H, m, H-1′), 5.71–5.69 (1H, m, H-3′), 5.79–5.77 (1H, m, H-3′′), 5.89–5.86 (1H, m, H-1′′), 6.09–6.07 (1H, m, H-2′), 6.25–6.22 (1H, m, H-2′′), 7.52 (1H, s, H-6). ^13^C NMR (CDCl_3_): 14.1 (CH_3_), 19.6, 22.6, 28.6, 29.0, 29.1 × 2, 31.8 ((CH_2_)_7_,), 37.3, 40.3 (C-5′, C-5′′), 56.8, 60.5 (C-1′, C-1′′), 71.4 (alkyne^α^), 74.5, 76.4 (C-4′, C-4′′), 95.1 (^β^alkyne), 100.9 (C-5), 130.7, 131.3 (C-2′, C-2′′), 137.1, 140.1 (C-3′, C-3′′), 141.6 (C-6), 150.6, 162.1 (C-4, C-2). HRMS: found *m/z* 413.2443; calcd for C_24_H_32_N_2_O_4_ [M + H] ^+^ 413.2435.

*1-[4′-Hydroxy-2′-cyclopenten-1′-yl]-3-(4′′-hydroxy-2′′-cyclopent-en-1′′-yl)-5-octynyluracil* (**4**)**.** Compound **4** was obtained similarly to the procedure described for **1**, with 1-octyne as a terminal alkyne. Purification (CHCl_3_/MeOH 98:2 then 95:5) and repurification (EtOAc/hexane 1:2) by PLC on silica gel afforded the title compound **4** as a white powder (40 mg, 52%). ^1^H NMR (CDCl_3_): 0.87–0.82 (3H, m, CH_3_), 1.26–1.23 (4H, m, (CH_2_)_2_), 1.38–1.33 (2H, m, CH_2_-c), 1.54–1.50 (2H, m, CH_2_-b), 1.62–1.58 (1H, m, Hb-5′), 1.96–1.90 (1H, m, Hb-5′′), 2.35–2.29 (2H, m, CH_2_-a), 2.78–2.68 (1H, m, Ha-5′), 2.90–2.80 (1H, m, Ha-5′′), 4.69–4.67 (1H, m, H-4′), 4.85–4.82 (1H, m, H-4′′), 5.53-5.51 (1H, m, H-1′), 5.71-5.68 (1H, m, H-3′), 5.79–5.77 (1H, m, H-3′′), 5.90–5.85 (1H, m, H-1′′), 6.09–6.07 (1H, m, H-2′), 6.24–6.21 (1H, m, H-2′′), 7.52 (1H, s, H-6). ^13^C NMR (CDCl_3_): 14.0 (CH_3_), 19.6, 22.5, 28.6, 29.7, 31.3 ((CH_2_)_5_,), 37.3, 40.3 (C-5′, C-5′′), 56.8, 60.5 (C-1′, C-1′′), 70.5 (alkyne^α^), 74.5, 76.2 (C-4′, C-4′′), 95.1 (^β^alkyne), 100.8 (C-5), 130.7, 131.3 (C-2′, C-2′′), 137.1, 140.1 (C-3′, C-3′′), 141.6 (C-6), 150.6, 162.1 (C-4, C-2). HRMS: found *m/z* 385.2132; calcd for C_22_H_28_N_2_O_4_ [M + H]^+^ 385.2122.

*1-[4′-Hydroxy-2′-cyclopenten-1′-yl]-3-(4′′-hydroxy-2′′-cyclopent-en-1′′-yl)-5-pentynyluracil* (**5**)**.** Compound **5** was obtained similarly to the procedure described for **1**, with 1-pentyne as a terminal alkyne. Purification (CHCl_3_/MeOH 98:2 then 95:5) and repurification (EtOAc/hexane 1:2) by PLC on silica gel afforded the title compound **5** as a white powder (40 mg, 56%). ^1^H NMR (CDCl_3_): 1.00–0.97 (3H, m, CH_3_), 1.57–1.55 (2H, m, CH_2_-b), 1.62–1.58 (1H, m, Hb-5′), 1.98–1.92 (1H, m, Hb-5′′), 2.35–2.31 (2H, m, CH_2_-a), 2.80–2.71 (1H, m, Ha-5′), 2.91–2.86 (1H, m, Ha-5′′), 4.71–4.69 (1H, m, H-4′), 4.87–4.85 (1H, m, H-4′′), 5.53 (1H, m, H-1′), 5.72–5.71 (1H, m, H-3′), 5.81–5.80 (1H, m, H-3′′), 5.91–5.89 (1H, m, H-1′′), 6.11–6.10 (1H, m, H-2′), 6.25–6.24 (1H, m, H-2′′), 7.53 (1H, s, H-6). ^13^C NMR (CDCl_3_): 13.7 (CH_3_), 21.6, 22.1 ((CH_2_)_2_,), 37.4, 40.4 (C-5′, C-5′′), 56.9, 60.6 (C-1′, C-1′′), 71.7 (alkyne^α^), 74.7, 76.3 (C-4′, C-4′′), 94.7 (^β^alkyne), 100.7 (C-5), 130.7, 131.5 (C-2′, C-2′′), 137.2, 140.1 (C-3′, C-3′′), 141.6 (C-6), 150.6, 162.1 (C-4, C-2). HRMS: found *m/z* 343.1657; calcd for C_19_H_22_N_2_O_4_ [M + H]^+^ 43.1652.

*1-[4′-Hydroxy-2′-cyclopenten-1′-yl]-3-(4′′-hydroxy-2′′-cyclopent-en-1′′-yl)-5-(phenylethenyl)uracil* (**6**). Compound **6** was obtained similarly to the procedure described for **1**, with 1-phenylacetylene as a terminal alkyne. Purification (CHCl_3_/MeOH 98:2 then 95:5) and repurification (EtOAc/hexane 1:2) by PLC on silica gel afforded the title compound **6** as a white powder (57 mg, 76%). ^1^H NMR (CDCl_3_): 1.63–1.57 (1H, m, Hb-5′), 1.98–1.91 (1H, m, Hb-5′′), 2.78–2.74 (1H, m, Ha-5′), 2.89-2.81 (1H, m, Ha-5′′), 3.51 and 4.06–4.01 (2H, 2m, HO-4′ and HO-4′′), 4.70–4.68 (1H, m, H-4′), 4.85–4.83 (1H, m, H-4′′), 5.57–5.53 (1H, m, H-1′), 5.73–5.71 (1H, m, H-3′), 5.79–5.78 (1H, m, H-3′′), 5.93–5.89 (1H, m, H-1′′), 6.12–6.10 (1H, m, H-2′), 6.27–6.25 (1H, m, H-2′′), 7.29–7.27 (3H, m, Ph), 7.46–7.43 (2H, m, Ph), 7.74 (1H, s, H-6). ^13^C NMR (CDCl_3_): 37.5, 40.7 (C-5′, C-5′′), 57.0, 60.7 (C-1′, C-1′′), 74.6, 76.4 (C-4′, C-4′′), 80.8 and 93.6 (alkyne), 100.4 (C-5), 122.8, 128.4 × 2, 128.7 (Ph), 130.7, 131.1 (C-2′, C-2′′), 131.8 × 2 (Ph), 137.4, 140.7 (C-3′, C-3′′), 142.5 (C-6), 150.7, 161.7 (C-4, C-2). HRMS: found *m/z* 377.1503; calcd for C_22_H_20_N_2_O_4_ [M + H]^+^ 377.1496.

*1-[4′-Hydroxy-2′-cyclopenten-1′-yl]-3-(4′′-hydroxy-2′′-cyclopent-en-1′′-yl)-5-(4-tertbutylphenynylethenyl)uracil* (**7**). Compound **7** was obtained similarly to the procedure described for **1**, with 4-tertbutylphenylacetylene as a terminal alkyne. Purification (CHCl_3_/MeOH 98:2 then 95:5) and repurification (EtOAc/hexane 1:2) by PLC on silica gel afforded the title compound **7** as a white powder (59 mg, 68%). ^1^H NMR (CDCl_3_): 1.28 (9H, s, CH_3_), 1.66–1.58 (1H, m, Hb-5′), 1.99–1.95 (1H, m, Hb-5′′), 2.80–2.72 (1H, m, Ha-5′), 2.92–2.84 (1H, m, Ha-5′′), 4.71–4.70 (1H, m, H-4′), 4.85 (1H, m, H-4′′), 5.54 (1H, m, H-1′), 5.74–5.72 (1H, m, H-3′), 5.79–5.78 (1H, m, H-3′′), 5.92–5.90 (1H, m, H-1′′), 6.11–6.10 (1H, m, H-2′), 6.26–6.25 (1H, m, H-2′′), 7.32–7.30 (2H, m, Ph), 7.40–7.38 (2H, m, Ph), 7.69 (1H, s, H-6). ^13^C NMR (CDCl_3_): 31.2 × 3, 34.9 (C(CH_3_)_3_), 37.4, 40.6 (C-5′, C-5′′), 56.9, 60.7 (C-1′, C-1′′), 74.6, 76.3 (C-4′, C-4′′), 80.0 and 93.9 (alkyne), 100.6 (C-5), 119.6, 125.4 × 2 (Ph), 130.8, 131.3 (C-2′, C-2′′), 131.5 × 2 (Ph), 137.2, 140.4 (C-3′, C-3′′), 142.1 (C-6), 150.6, 152.9, 161.7 (C-4, C-4Ph, C-2). HRMS: found *m/z* 433.2129; calcd for C_26_H_28_N_2_O_4_ [M + H]^+^ 433.2122.

*1-[4′-Hydroxy-2′-cyclopenten-1′-yl]-3-(4′′-hydroxy-2′′-cyclopent-en-1′′-yl)-5-(4-pentyl-phenylethenyl)uracil* (**8**). Compound **8** was obtained similarly to the procedure described for **1**, with 4-pentylphenylacetylene as a terminal alkyne. Purification (CHCl_3_/MeOH 98:2 then 95:5) and repurification (EtOAc/hexane 1:2) by PLC on silica gel afforded the title compound **8** as a white powder (59 mg, 66%).^1^H NMR (CDCl_3_): 0.87–0.83 (3H, m, CH_3_), 1.33–1.25 (4H, m, (CH_2_)_2_), 1.59–1.54 (2H, m, CH_2_-b), 1.65–1.59 (1H, m, Hb-5′), 1.98–1.93 (1H, m, Hb-5′′), 2.58–2.53 (2H, m, CH_2_-a), 2.81–2.70 (1H, m, Ha-5′), 2.92–2.86 (1H, m, Ha-5′′), 4.71–4.69 (1H, m, H-4′), 4.87–4.85 (1H, m, H-4′′), 5.56–5.52 (1H, m, H-1′), 5.73–5.71 (1H, m, H-3′), 5.81–5.79 (1H, m, H-3′′), 5.93–5.88 (1H, m, H-1′′), 6.12–6.10 (1H, m, H-2′), 6.27–6.24 (1H, m, H-2′′), 7.11–7.08 (2H, m, Ph), 7.38–7.35 (2H, m, Ph), 7.68 (1H, s, H-6). ^13^C NMR (CDCl_3_): 14.0 (CH_3_), 22.5, 30.8, 31.4, 35.9 ((CH_2_)_4_), 37.3, 40.5 (C-5′, C-5′′), 56.8, 60.6 (C-1′, C-1′′), 74.6, 76.3 (C-4′, C-4′′), 79.9 and 93.8 (alkyne), 100.5 (C-5), 119.7, 128.4 × 2 (Ph), 130.6, 131.2 (C-2′, C-2′′), 131.6 × 2 (Ph), 137.2, 140.3 (C-3′, C-3′′), 141.9 (C-6), 143.8 (Ph), 150.5, 161.5 (C-4, C-2). HRMS: found *m/z* 447.2286; calcd for C_27_H_30_N_2_O_4_ [M + H]^+^ 447.2278.

*1-[4′-Hydroxy-2′-cyclopenten-1′-yl]-3-(4′′-hydroxy-2′′-cyclopent-en-1′′-yl)-5-(hydroxypropynyl)uracil* (**9**)**.** Compound **9** was obtained similarly to the procedure described for **1**, with propargyl alcohol as an alkyne. Purification (CHCl_3_/MeOH 98:2 then 95:5) and repurification (EtOAc/hexane 1:2) by PLC on silica gel afforded the title compound **9** as a white powder (41 mg, 62%).^1^H NMR (DMSO-d6): 1.48–1.40 (1H, m, Hb-5′), 1.96–1.90 (1H, m, Hb-5′′), 2.54–2.48 (1H, m, Ha-5′), 2.77-2.71 (1H, m, Ha-5′′), 4.25 and 4.23 (2H, 2s, HO-4′ and HO-4′′), 4.63–4.61 (2H, m, H-4′ and OH), 4.76–4.74 (1H, m, H-4′′), 5.29–5.25 (1H, m, H-1′), 5.33–5.29 (1H, m, H-3′), 5.45–5.43 (1H, m, H-3′′), 5.63–5.58 (1H, m, H-1′′), 5.80 (2H, s, CH_2_), 5.85–5.83 (1H, m, H-2′), 6.20–6.18 (1H, m, H-2′′), 7.72 (1H, s, H-6). ^13^C NMR (DMSO-d6): 28.9 (CH_2_), 37.4, 49.4 (C-5′, C-5′′), 56.1, 59.7 (C-1′, C-1′′), 73.3, 74.0 (C-4′, C-4′′), 76.4 and 92.4 (alkyne), 97.7 (C-5), 130.5, 131.8 (C-2′, C-2′′), 134.2, 140.6 (C-3′, C-3′′), 143.4 (C-6), 149.8, 161.2 (C-4, C-2). HRMS: found *m/z* 331.1293; calcd for C_17_H_18_N_2_O_5_ [M + H]^+^ 331.1288.

*1-[4′-Hydroxy-2′-cyclopenten-1′-yl]-3-(4′′-hydroxy-2′′-cyclopent-en-1′′-yl)-5-(trifluoroacetamidepropynyl)uracil* (**10**)**.** Compound **10** was obtained similarly to the procedure described for **1**, with 2,2,2-trifluoro-N-(prop-2-yn-1-yl)acetamide as an alkyne. Purification (CHCl_3_/MeOH 98:2 then 95:5) and repurification (EtOAc/hexane 1:2) by PLC on silica gel afforded the title compound **10** as a yellow powder (61 mg, 72%). ^1^H NMR (CD_3_OD:CDCl_3_): 1.51–1.44 (1H, m, Hb-5′), 1.96–1.91 (1H, m, Hb-5′′), 2.79–2.68 (1H, m, Ha-5′), 2.92–2.82 (1H, m, Ha-5′′), 4.21 (2H, s, CH_2_), 4.69–4.67 (1H, m, H-4′), 4.76–4.74 (1H, m, H-4′′), 5.53 (1H, m, H-1′), 5.76 (2H, m, H-3′ and H-3′′), 5.85–5.83 (1H, m, H-1′′), 6.01 (1H, m, H-2′), 6.23 (1H, m, H-2′′), 7.45 (1H, br.s, NH), 7.77 (1H, s, H-6). ^13^C NMR (CD_3_OD:CDCl_3_): 29.3 (CH_2_), 36.7, 39.9 (C-5′, C-5′′), 56.7, 60.4 (C-1′, C-1′′), 73.9, 75.3 (C-4′, C-4′′), 74.7 and 86.9 (alkyne), 98.1 (C-5), 119.0 (CF3), 130.5, 131.7 (C-2′, C-2′′), 134.4, 140.0 (C-3′, C-3′′), 143.9 (C-6), 150.4, 157.9, 162.6 (C-4, C(O)NH, C-2). HRMS: found *m/z* 426.1305; calcd for C_19_H_18_F_3_N_3_O_5_ [M + H]^+^ 426.1291.

*1-[4′-Hydroxy-2′-cyclopenten-1′-yl]-3-(4′′-hydroxy-2′′-cyclopent-en-1′′-yl)-5-(ethyloxycarbonylpropynyl)uracil* (**11**)**.** Compound **11** was obtained similarly to the procedure described for **1**, with ethyl propiolate as an alkyne. Purification (CHCl_3_/MeOH 98:2 then 95:5) and repurification (EtOAc/hexane 1:2) by PLC on silica gel afforded the title compound **11** as a yellow powder (53 mg, 71%). ^1^H NMR (CDCl_3_): 1.32–1.27 (3H, m, CH_3_), 1.70–1.58 (1H, m, Hb-5′), 1.98–1.92 (1H, m, Hb-5′′), 2.79–2.72 (1H, m, Ha-5′), 2.92–2.82 (1H, m, Ha-5′′), 4.27–4.19 (2H, m, CH_2_), 4.72–4.69 (1H, m, H-4′), 4.87–4.83 (1H, m, H-4′′), 5.59-5.57 (1H, m, H-1′), 5.72–5.69 (1H, m, H-1′′), 5.87–5.83 (2H, m, H-3′ and H-3′′), 6.13–6.11 (1H, m, H-2′), 6.30–6.29 (1H, m, H-2′′), 7.93–7.91 (1H, m, H-6). ^13^C NMR (CDCl_3_): 14.0 (CH_3_), 37.2, 40.4 (C-5′, C-5′′), 57.1, 61.0 (C-1′, C-1′′), 62.2 (CH_2_), 74.4, 76.2 (C-4′, C-4′′), 76.4 and 84.9 (alkyne), 99.6 (C-5), 130.3, 131.1 (C-2′, C-2′′), 137.5, 140.7 (C-3′, C-3′′), 146.7 (C-6), 150.3, 153.6, 161.2 (C-4, C(O)O, C-2). HRMS: found *m/z* 373.1403; calcd for C_19_H_20_N_2_O_6_ [M + H]^+^ 373.1394.

### 4.2. Antiparasite Experiments

#### 4.2.1. Parasite Strains and Cultures

##### *Trypanosoma brucei brucei* 

The standard drug sensitive strain *T. b. brucei* strain Lister 427 (s427-WT) was used as reference strain [37]. B48 was derived by knockout of the drug transporter TbAT1 [23] and subsequent in vitro adaptation to very high levels of resistance to pentamidine, diminazene, and melaminophenyl arsenicals [20,21]. 5FURes was derived from s427WT by in vitro adaptation to high concentrations of 5-fluorouracil [12]. PYR6-5-/- was created by the knockout of the URA6-5 open reading frame from *T. b. brucei* strain 2T1, itself derived from s427WT [13]. All strains were grown as bloodstream forms, in HMI-9 medium containing 10% fetal bovine serum (FBS; Gibco, Paisley, UK), 14 µL/L β-mercaptoethanol, and 3 g/L NaHCO_3_, pH adjusted to 7.4 as described [38] at 37 °C/5% CO_2_.

##### *Leishmania mexicana* 

Promastigotes of *Leishmania mexicana* strain MNY/BZ/62/M379 was cultured in a minimal essential medium, HO-MEMO-MEME, supplemented with 10% FBS and 1% of a penicillin/streptomycin solution (Gibco) as described previously [39] at 25 °C. The *L. mexicana* 5FURes strain was the same strain but adapted in vitro to high levels of 5FU exactly as described for *T. brucei* [12].

##### *Trichomonas vaginalis* 

The standard metronidazole-susceptible *T. vaginalis* strain G3was originally obtained from Jeremy Mottram (University of York, UK) and cultured in a modification of Diamond′s medium supplemented with 10% horse serum with a composition as described [40] at 37 °C under anaerobic conditions. Cultures were passaged daily. 

#### 4.2.2. Determination of EC_50_ Values for Protozoan Parasites

##### *Trypanosoma brucei* 

EC_50_ values were determined using our standardized resazurin assay [41], based on the reduction of blue, non-fluorescent resazurin sodium salt (Sigma) to pink, fluorescent resorufin [42]. Briefly, the assay was performed in 96-well plates with 11 doubling dilutions of the test compounds, starting at 100 µM. To each well 2 × 10^4^ bloodstream form trypanosomes were added, and the plates were incubated 48 h at 37 °C/5% CO_2_ at which point 20 µL of resazurin solution was added and the plates incubated for another 24 h. Fluorescence was read on a FLUOstar Optima (BMG Labtech plate reader and the data fitted to a sigmoid curve with variable slope using Prism 6.0 GraphPad, San Diego, CA, USA). 

##### *Leishmania mexicana* 

The resazurin assay for *L. mexicana* promastigotes was performed exactly as for *T. b. brucei*, except that incubation times of 72 and 48 h were used before and after the addition of resazurin, respectively, due to the slower metabolism of the dye by *Leishmania promastigotes* [42]. 

##### *Trichomonas vaginalis* 

Drugs were tested on *T. vaginalis* using a resorufin-based assay that we developed [40,43]. The assay is based on the principle that live *T. vaginalis* trophozoites are, almost uniquely, able to reduce fluorescent, pink resorufin to non-fluorescent, colorless dihydro-resorufin. This conversion was measured as a dose-dependent increase in fluorescence (with dead cells unable to metabolize the fluorophore; λ_exc_ 544 nm, λ_em_ 599 nm), resulting in a sigmoid curve with variable slope that yielded EC_50_ values (Prism 6.0). Plates contained 11 doubling dilutions (1 row) and no-drug control for each compound starting at 100 µM under anaerobic conditions as described [40,43]. Fluorescence was determined in a PheraStar plate reader (BMG Labtech).

### 4.3. Antibacterial Experiments

#### 4.3.1. Bacterial Strains

The *Mycobacterium tuberculosis* ATCC 25,177, *Mycobacterium bovis* ATCC 35,737, *Mycobacerium smegmatis* ATCC 700,084, *Pseudomonas aeruginosa* ATCC 27,853, and *Escherichia coli* ATCC 25,922 strains used in these studies were purchased from the American Type Culture Collection (Manassas, VA, USA). *Staphylococcus aureus* strain NRS384 was obtained from BEI Resources. The bacteria were propagated as recommended by the supplier. The virulent laboratory strain of an *M. tuberculosis* H37Rv susceptible to anti-TB drugs and a clinical MDR strain MS-115 were used. The MDR strain is resistant toward five anti-TB drugs of the first line: rifampicin, isoniazid, streptomycin, ethambutol, and pyrazinamide. Single cells suspension of Mycobacteria at the same growth phase was normalized by CFU [42]. The enriched Dubois medium (Difco, Detroit, MI, USA) was used. 

#### 4.3.2. Determination of Minimal Inhibitory Concentration Against *M. tuberculosis* ATCC 25,177

The susceptibility of *M. tuberculosis* ATCC 25,177 to the test compounds was evaluated by determining the MIC of each compound using a micro-broth dilution analysis. A standardized inoculum was prepared by direct suspension of bacteria from a Lowenstein–Jensen slant to Middlebrook 7H9 broth with ADC (albumin, dextrose, catalase) enrichment. The broth culture was incubated for 26 days at 37 °C. Following the incubation, the culture was vortexed for approximately 1 min with 1.0 mm glass beads (enough to cover the bottom of the tube) resulting in an OD595 of 0.18 and then further diluted 1:20 to yield 5 × 106 CFU/mL. A total of 100 μL was added to triplicate wells of a 96-well plate containing 100 μL of test compound serially diluted 2-fold in the appropriate broth. This dilution scheme yielded final concentrations of the bacteria estimated to be 2.5 × 106 CFU/mL. The plates were incubated for 7 days at 37 °C at which time 30 µL of resazurin solution at 0.01% was added to each well and the cultures were incubated for an additional 24 h at 37 °C. Following the incubation, the plates were visually scored +/− for color change from blue (no growth) to pink (growth). The MIC for each compound was determined as the lowest compound dilution that completely inhibited microbial growth. 

#### 4.3.3. Determination of Minimal Inhibitory Concentration Against *M. bovis* ATCC 35,737

The susceptibility of *M. bovis* ATCC 35,737 to the test compounds was evaluated by determining the MIC of each compound using a micro-broth dilution. A standardized inoculum was prepared by direct suspension of bacteria from a Lowenstein–Jensen slant to Middlebrook 7H9 broth with ADC (albumin, dextrose, catalase) enrichment and 40 mM sodium pyruvate. The broth culture was incubated for 23 days at 37 °C. Following the incubation, the culture was vortexed for 1 min with 1.0. mm glass beads (enough to cover the bottom of the tube) as above, resulting in an OD_595_ of 0.203 which was further diluted 1:10 to yield 3 × 10^7^ CFU/mL. A total of 100 μL was added in triplicate wells of a 96-well plate containing 100 μL of test compound serially diluted 2-fold in the appropriate broth. This dilution scheme yielded final concentrations of the bacteria estimated to be 1.5 × 10^7^ CFU/mL. The plates were incubated for 5 days at 37 °C at which time 30 µL of a 0.01% resazurin solution was added to each well. The cultures were incubated for an additional 4 days at 37 °C at which time the plates were visually scored +/− for color change from blue (no growth) to pink (growth). The MIC for each compound was determined as the lowest compound dilution that completely inhibited microbial growth.

#### 4.3.4. Antituberculosis Tests with Virulent Strains of *M. tuberculosis*

The automated BACTEC MGIT 960 system (BD, city state abbreviation, Westfield, MA, USA) was used [44]. Tested compounds were dissolved in DMSO at a concentration of 40 mg/mL (stock solutions). Water and Twin-80 were added to the stock solutions to provide DMSO/Twin-80/H2O (5:0.5:4.5, *v/v/v).* The concentration of mycobacterial cells was 106 CFU/mL. Each sample including the control samples lacking the tested compound were studied in triplicate. The antibacterial activity was evaluated with the TB Exist software [45]. The method is based on comparison of the bacterial growth in the samples, containing the tested compounds, and the control samples diluted 100 times if compared with the initially inoculated culture. The culture is susceptible (that is, the compound is active) if the optical density of the control sample is 400 GU (growth units) and that of the tested sample is less than 100 GU. In addition, using the absolute concentrations method a delay in the bacterial growth of the tested sample was measured in comparison with the control samples, which reflected a negative effect of the tested compounds at the concentrations lower than MIC on the bacterial viability. The measurements were carried out automatically every 1 h and registered with the Epicenter Software (BD, city state abbreviation, Westfield, MA, USA).

### 4.4. Evaluation of Cytotoxicity

#### 4.4.1. U-937 Cells

The U-937 cells used in the cytotoxicity evaluation were obtained from the AIDS Research and Reference Program. The cells were cultured as indicated by the supplier in RPMI-1640 supplemented with 10% heat inactivated FBS, 1% penicillin/streptomycin solution, and 1% l-glutamine. 

#### 4.4.2. Evaluation of U-937 Cytotoxicity 

U-937 cells plated at 2.5 × 10^3^ cells/well in a volume of 100 µL were incubated with serially diluted test compounds in a volume of 100 µL for 6 days at 37 °C/5% CO_2_. Following the incubation, the cells were stained by adding 50 μL tetrazolium dye XTT (Sigma) solution at 5 mg/mL to determine cellular viability and read at 460/650 nm on a spectrophotometer. Toxicity values were calculated using linear regression analysis.

## 5. Conclusions

This second generation series of compounds displayed moderate activity against kinetoplastid parasites but at best, only marginal activity against *Trichomonas vaginalis* and bacteria. The alkyne containing sidechain from the 5-position of the pyrimidine ring is most likely required for uptake via passive diffusion. Further optimization should be possible through exploration of the SAR of the pyrimidine pharmacophore and the identification of the cellular target. Additional studies will be underway soon and the results of those reported elsewhere as they become available. 

## Figures and Tables

**Table 1:** Antitrypanosomal results.

Compound	*T. Brucei*, EC_50_, µM				U-937 Cells
	S427-WT	B48	2T1 PYR6-5 ^−/−^	Tbb-5FURes	TC_50_, µM
**1**	8.0 ± 0.1	9.0 ± 0.1	9.2 ± 0.4	7.5 ± 0.1	66.3
**2**	3.8 ± 0.1	4.0 ± 0.4	5.5 ± 0.5 *	5.2 ± 0.6	22.1
**3**	13.2 ± 0.6	14.7 ± 0.2	17.1 ± 1.2 *	19.9 ± 0.7 ***	2.2
**4**	13.6 ± 0.1	17.7 ± 1.0 *	17.5 ± 1.2 *	24.9 ± 0.5 ***	73.8
**5**	NE ^1^	NE	NE	NE	88.5
**6**	NE	NE	NE	NE	166.2
**7**	8.7 ± 0.4	10.8 ± 0.6 *	14.7 ± 0.4 ***	12.6 ± 1.4	22.5
**8**	7.8 ± 0.3	11.0 ± 0.6 *	15.4 ± 0.7 ***	12.0 ± 1.2 *	17.3
**9**	66.8 ± 6.6	76.8 ± 4.4 **	59.9 ± 1.0	74.9 ± 3.5	>100
**10**	NE	NE	NE	NE	78.0
**11**	NE	NE	NE	NE	21.6
5-Fluorouracil	87 ± 6.4	101 ± 7	2.33 ± 0.35 ***	2497 ± 76 ***	ND ^2^
Pentamidine	0.0032 ± 0.0004	0.71 ± 0.03 ***	0.0031 ± 0.0004	0.0034 ± 0.0004	ND
N9	ND	ND	ND	ND	6.5 µg/mL

NE, no effect on cell viability at 100 µM. ND, not determined * *p* < 0.05; ** *p* < 0.01; *** *p* < 0.001, by Student’s unpaired, two-tailed *t*-test. All numbers are average and SEM of at least three independent determinations.

**Table 2:** Activity against *Leishmania mexicana* and *Trichomonas vaginalis*.

Compound	*L. Mexicana* WT, EC_50_, µM	*L. Mexicana* 5FUR, EC_50_, µM	*Trichomonas Vaginalis*, EC_50_, µM
**1**	11.8 ± 0.3	11.4 ± 0.3	ND
**2**	28.3 ± 3.0	51.1 ± 0.6 **	48.9 ± 0.05
**3**	13.8 ± 1.7	25.8 ± 0.4 ***	32.0 ± 0.001
**4**	45.6 ± 0.9	47.3 ± 0.4	54.1 ± 0.002
**5**	>100	>100	73.5 ± 0.08
**6**	NE ^1^	NE	70.2 ± 0.06
**7**	21.1 ± 2.9	45.3 ± 2.2 ***	52.8 ± 0.007
**8**	13.0 ± 1.5	27.5 ± 2.3 **	36.9 ± 0.18
**9**	NE	NE	84.8 ± 0.004
**10**	NE	NE	69.2 ± 0.05
**11**	NE	NE	53.8 ± 0.74
5-Fluorouracil	8.7 ± 2.1	3516 ± 440 ***	ND
Pentamidine	1.2 ± 0.8	1.63 ± 0.18	ND
Metronidazole	ND	ND	0.53 ± 0.10

NE, no effect on cell viability at 100 µM. * *p* < 0.05; ** *p* < 0.01; *** *p* < 0.001, by Student’s unpaired, two-tailed *t*-test. All numbers are average and SEM of at least three independent determinations. ND, not determined.

**Table 3:** Preliminary evaluation of antimycobacterial activity of compounds **1**–**11.**

Compound	*M. Tuberculosis 25,177*	
	MIC, µM	MIC, µg/mL
**1**	250	117.3
**2**	31	13.6
**3**	31	12.8
**4**	31	11.9
**5**	>1000	>342.4
**6**	500	188.2
**7**	62.5	27.0
**8**	31	13.9
**9**	1000	330.3
**10**	>1000	>425.4
**11**	1000	372.4
DMSO, %	>5	>0.05
Kanamycin	6.5	3.13
Rifampicin	0.19	0.16

**Table 4:** Activity 5′-Norcarbocyclic nucleoside analogues against virulent *M. tuberculosis* strains.

Compound	*M. Tuberculosis H37Rv* MIC, µg/mL	*M. Tuberculosis MS-115* MIC, µg/mL
**1**	50	>50
**2**	>50	ND
**3**	50	50
**4**	>50	ND
**7**	>50	ND
**8**	>50	ND
Rifampicin	1	>50
Isoniazid	0.1	>100
Levofloxacin	1.5	1.5
